# *Teichospora* and the Teichosporaceae

**DOI:** 10.1007/s11557-016-1171-2

**Published:** 2016-03-03

**Authors:** Walter M. Jaklitsch, Ibai Olariaga, Hermann Voglmayr

**Affiliations:** Institute of Forest Entomology, Forest Pathology and Forest Protection, Department of Forest and Soil Sciences, BOKU-University of Natural Resources and Life Sciences, Hasenauerstraße 38, 1190 Vienna, Austria; Division of Systematic and Evolutionary Botany, Department of Botany and Biodiversity Research, University of Vienna, Rennweg 14, 1030 Wien, Austria; Museum of Evolution, Uppsala University, Norbyvägen 16, SE-75236 Uppsala, Sweden

**Keywords:** *Ascomycota*, *Cucurbitaria*, Phylogenetic analysis, Pleosporales, *Strickeria*, *Teichosporella*

## Abstract

A multigene analysis of a combined ITS, LSU, SSU, *rpb2* and *tef1* sequence data matrix was applied to infer the phylogenetic position of the genus *Teichospora* in the Pleosporales, based on isolates from freshly collected material of the generic type *T. trabicola* and several additional species. Phylogenetic analyses revealed that *Misturatosphaeria* and *Floricola* are synonyms of *Teichospora*. All species of these genera and several species recently described in the genus *Curreya* belong to *Teichospora* and are thus combined in this genus. Also, *Melanomma radicans* and *Ramusculicola thailandica* are combined in *Teichospora*. The new name *Teichospora parva* is established for *Misturatosphaeria minima*. Three new species, *T. melanommoides*, *T. pusilla* and *T. rubriostiolata*, are described, and an expanded description of *T. mariae* is given. The family Teichosporaceae is currently confined to *Teichospora*, which can be phylogenetically clearly separated from *Lophiostoma*, the type genus of the Lophiostomataceae. The family name Floricolaceae is a synonym of Teichosporaceae. All species described here form apically free paraphyses among immature asci. This finding contradicts the current general dogma that apically free paraphyses are absent in the Pleosporales and questions the wide use of the term pseudoparaphysis.

## Introduction

In the Pleosporales, sexual morphs with brown muriform ascospores are particularly difficult to classify. The types of many genera have not been recollected and sequenced, and several new genera have been described without sufficient knowledge of the limits of existing genera. Particularly important in this respect are species-rich genera. One of these is *Teichospora*, for which Index Fungorum lists 267 epithets. Many were combined in other genera such as *Chaetoplea*, *Cucurbitaria*, *Pleospora*, *Strickeria* and others (Barr 1990; see also links to Species Fungorum in Index Fungorum under *Teichospora*). In foregoing papers, we have determined the phylogenetic position of the generic types of *Cucurbitaria* (Doilom et al. 2013) and *Strickeria* (Jaklitsch et al. 2016). The latter belongs to the Xylariales and is thus unavailable for dothideomycetous fungi. However, 467 names are listed in *Cucurbitaria*, and 197 epithets in *Strickeria*, making clear that substantial efforts are still needed for species reclassification. In this paper, we provide the basis for the classification of *Teichospora* and the Teichosporaceae. Fuckel (1870) typified his genus *Teichospora* with *T. trabicola*. In the protologue of this species he gave the detailed information that the fungus occurs on poles in vineyards, the habitat where we primarily collected *Teichospora*. Since then, most wooden poles have been replaced by metal ones, but some are still made of wood. In Fuckel’s original area, these are now made of chemically treated coniferous wood, on which only lichens occur abundantly, and poles of *Robinia pseudoacacia*. Poles of the latter host, standing and lying on the ground, were examined for pyrenomycetous fungi and four species of *Teichospora* were found on them. The results are comparable with those obtained from poles in vineyards present around Vienna, Austria.

In the family Teichosporaceae, Barr (2002) assembled an apparently heterogeneous group of genera, which she separated from her earlier circumscribed Dacampiaceae (Barr 1987) on the basis of different trophic states (saprobic vs. lichenicolous in *Dacampia*), peridium and ascus structure. Apart from the type genus *Teichospora*, she placed the genera *Bertiella*, *Byssothecium*, *Chaetomastia*, *Immotthia*, *Loculohypoxylon*, *Moristroma*, and *Sinodidymella* in the Teichosporaceae. *Moristroma* was referred to the Chaetothyriomycetidae by Nordén et al. (2005), *Byssothecium* to the Massarinaceae (Schoch et al. 2009) and *Bertiella* to the Melanommataceae (Mugambi and Huhndorf 2009), but none of the remaining genera including *Teichospora* has been sequenced, and thus their phylogenetic position is unknown. Here, we redescribe the generic type of *Teichospora*, *T. trabicola*, and cement its phylogenetic position.

During preparation of our manuscript, Thambugala et al. (2015) published an account of the Lophiostomataceae, in which they proposed the new family Floricolaceae containing *Misturatosphaeria* and seven newly segregated small genera. Here, we synonymise Floricolaceae with Teichosporaceae and all newly introduced genera with *Teichospora*, based on arguments that include molecular phylogenetic considerations and morphology.

## Materials and methods

### Isolates and specimens

All newly prepared isolates used in this study originated from ascospores of fresh specimens. Numbers of strains including NCBI GenBank accession numbers of gene sequences used to compute the phylogenetic trees are listed in Table 1. Strain acronyms other than those of official culture collections are used throughout this work primarily as strain identifiers. Representative isolates have been deposited at the CBS-KNAW Fungal Biodiversity Centre, Utrecht, The Netherlands (CBS). Details of the specimens used for morphological investigations are listed in the Taxonomy section under the respective descriptions. Herbarium acronyms are according to Thiers (2015). Specimens have been deposited in the Herbarium of the Institute of Botany, University of Vienna (WU).

**Table 1:** Fungal names, strains and GenBank accessions used in this study

Taxon	Strain/specimen	ITS	LSU	SSU	*rpb2*	*tef1*
*Biatriospora marina*	CY 1228		GQ925848	GQ925835	GU479823	GU479848
*Byssosphaeria jamaicana*	SMH 1403		GU385152			GU327746
*Byssosphaeria rhodomphala*	GKM L153N		GU385157			GU327747
*Byssosphaeria salebrosa*	SMH 2387		GU385162			GU327748
*Byssosphaeria schiedermayeriana*	GKM 1197		GU385161			GU327750
*Byssosphaeria villosa*	GKM 204N		GU385151			GU327751
*Decaisnella formosa*	BCC 25617		GQ925847	GQ925834	GU479824	GU479850
*Halotthia posidoniae*	BBH 22481		GU479786	GU479752		
*Herpotrichia diffusa*	CBS 250.62		DQ678071	GU205239	DQ677968	DQ677915
*Herpotrichia juniperi*	CBS 200.31		DQ678080	DQ678029	DQ677978	DQ677925
*Herpotrichia macrotricha*	GKM 196N		GU385176			GU327755
*Lophiostoma arundinis*	CBS 621.86	AJ496633	DQ782384	DQ782383	DQ782386	DQ782387
*Lophiostoma caulium*	CBS 623.86		GU301833	GU296163	GU371791	
*Lophiostoma compressum*	KT 534	JN942962	JN941379	JN941376	JN993492	
*Lophiostoma crenatum*	CBS 629.86		DQ678069	DQ678017	DQ677965	DQ677912
*Lophiostoma fuckelii*	CBS 101952		DQ399531	FJ795496	FJ795472	
*Lophiostoma macrostomoides*	CBS 123097		FJ795439	FJ795482	FJ795458	GU456277
*Lophiostoma macrostomum*	KT 508	JN942961	AB619010	AB618691	JN993491	
*Lophiostoma quadrinucleatum*	GKM 1233		GU385184			GU327760
*Lophiostoma sagittiforme*	HHUF 29754	NR_119393	NG_042319	AB618693		
*Lophiostoma scabridisporum*	BCC 22835		GQ925844	GQ925831	GU479830	GU479857
*Lophiostoma triseptatum*	SMH 5287		GU385187			
*Mauritiana rhizophorae*	BCC 28866		GU371824	GU371832		GU371817
*Melanomma pulvis-pyrius*	CBS 124080		GU456323	GU456302	GU456350	GU456265
*Monotosporella tuberculata*	CBS 256.84		GU301851			GU349006
*Preussia funiculata*	CBS 659.74		GU301864	GU296187	GU371799	GU349032
*Preussia lignicola*	CBS 264.69		GU301872	GU296197	GU371765	GU349027
*Preussia minima*	CBS 524.50		DQ678056	DQ678003	DQ677950	DQ677897
*Preussia terricola*	DAOM 230091		NG_027612	AY544726	DQ470895	DQ471063
*Prosthemium betulinum*	CBS 279.74		DQ678078	DQ678027	DQ677976	DQ677923
*Roussoella hysterioides*	KT 1651		AB524621	AB524480	AB539101	AB539114
*Roussoella pustulans*	KT 1709		AB524623	AB524482	AB539103	AB539116
*Roussoellopsis tosaensis*	KT 1659		AB524625	AB524484	AB539104	AB539117
*Teichospora acaciae*	CPC 24801	KR611877	KR611898			
*Teichospora aurantiacinotata*	GKM 1238		GU385173			GU327761
*Teichospora aurantiacinotata*	GKM 1280		GU385174			GU327762
*Teichospora austroafricana*	CBS 119330		EU552115			
*Teichospora austroafricana*	CBS 122674		EU552116			
*Teichospora claviformis*	GKM 1210		GU385212			GU327763
*Teichospora cruciformis*	SMH 5151		GU385211			
*Teichospora grandicipis*	CPC 1852	JN712456	JN712520			
*Teichospora grandicipis*	CPC 1853	JN712457	JN712521			
*Teichospora kenyensis*	GKM 1195		GU385194			GU327767
*Teichospora kenyensis*	GKM 194N					GU327764
*Teichospora kenyensis*	GKM 234N		GU385188			GU327765
*Teichospora kenyensis*	GKM L100Na		GU385189			GU327766
*Teichospora mariae*	C134m	KU601580	KU601580			KU601614
*Teichospora mariae*	C136	KU601581	KU601581		KU601595	KU601611
*Teichospora mariae*	C139	KU601582	KU601582			KU601615
*Teichospora mariae*	C144	KU601583	KU601583			KU601613
*Teichospora mariae*	C159	KU601584	KU601584			KU601612
*Teichospora mariae*	CBS 124079		JN851819			KR075166
*Teichospora melanommoides*	MP5	KU601585	KU601585			KU601610
*Teichospora parva*	ANM 60		GU385182			
*Teichospora parva*	ANM 933		GU385195			
*Teichospora parva*	GKM 169N		GU385165			GU327768
*Teichospora parva*	SMH 2448		GU385166			
*Teichospora proteae*	CBS 122675		EU552117			
*Teichospora pusilla*	C140	KU601586	KU601586			KU601605
*Teichospora radicans*	SMH 4330		GU385167			GU327770
*Teichospora radicans*	ATCC 42522		U43479	U43461	AY485625	
*Teichospora rubriostiolata*	C158	KU601587	KU601587		KU601596	KU601607
*Teichospora rubriostiolata*	C158x	KU601588	KU601588		KU601597	KU601608
*Teichospora rubriostiolata*	TR5	KU601589	KU601589		KU601598	KU601606
*Teichospora rubriostiolata*	TR7	KU601590	KU601590		KU601599	KU601609
*Teichospora* sp.	MFLUCC 12-0088		KF531927	KF531928		
*Teichospora* sp.	SMH 3747		GU385196			
*Teichospora striata*	JK 5603K		GU479785	GU479751		
*Teichospora striata*	JK 5678I		GU301813	GU296149	GU371758	GU479852
*Teichospora viticola*	IT-2178	KT305997	KT305993	KT305995		
*Teichospora tennesseensis*	ANM 911		GU385207			GU327769
*Teichospora thailandica*	MFLUCC 13-0284	KP899141	KP888647	KP899131		KR075167
*Teichospora thailandica*	MFLUCC 10-0126	KP899138	KP888644	KP899130		KR075170
*Teichospora trabicola*	C134	KU601591	KU601591		KU601600	KU601601
*Teichospora trabicola*	C141	KU601592	KU601592			KU601603
*Teichospora trabicola*	C157	KU601593	KU601593			KU601604
*Teichospora trabicola*	C160	KU601594	KU601594			KU601602
*Teichospora uniseriata*	ANM 909		GU385206			
*Westerdykella angulata*	CBS 610.74		DQ384105	DQ384067		GU371821
*Westerdykella cylindrica*	CBS 454.72		AY004343	AY016355		GU349021
*Westerdykella dispersa*	CBS 508.75		DQ384099			
*Westerdykella ornata*	CBS 379.55		GU301880	GU296208	GU371803	

### Culture preparation, growth rate determination and phenotype analysis

Cultures were prepared and maintained as described previously (Jaklitsch 2009). Microscopic observations were made in tap water except where noted. Morphological analyses of microscopic characters were carried out as described earlier (Jaklitsch 2009). Methods of microscopy included stereomicroscopy using a Nikon SMZ 1500 and Nomarski differential interference contrast (DIC) using the compound microscope Nikon Eclipse E600. Images and data were gathered using a Nikon Coolpix 4500 or a Nikon DS-U2 digital camera and measured with NIS-Elements D v.3.0. Measurements are reported as maximum and minimum in parentheses and the mean plus and minus the standard deviation of a number of measurements given in parentheses.

### DNA extraction and sequencing methods

The extraction of genomic DNA was performed as reported previously (Voglmayr and Jaklitsch 2011; Jaklitsch et al. 2012) using the DNeasy Plant Mini Kit (Qiagen, Hilden, Germany). The following loci were amplified and sequenced: the complete internally transcribed spacer region (ITS1-5.8S-ITS2) and a c.900-bp fragment of the large subunit nuclear ribosomal DNA (nLSU rDNA), amplified and sequenced as a single fragment with primers V9G (de Hoog and Gerrits van den Ende 1998) and LR5 (Vilgalys and Hester 1990); a c.1.2-kb fragment of the RNA polymerase II subunit 2 (*rpb2*) with primers fRPB2-5f and fRPB2-7cr (Liu et al. 1999); and a c.1.3-kb fragment of the translation elongation factor 1-alpha (*tef1*) with primers EF1-728F (Carbone and Kohn 1999) and TEF1LLErev (Jaklitsch et al. 2005). PCR products were purified using an enzymatic PCR cleanup (Werle et al. 1994) as described in Voglmayr and Jaklitsch (2008). DNA was cycle-sequenced using the ABI PRISM Big Dye Terminator Cycle Sequencing Ready Reaction Kit v.3.1 (Applied Biosystems, Warrington, UK) with the same primers as in PCR. In addition, the primers ITS4 (White et al. 1990) and LR3 (Vilgalys and Hester 1990) were used for the ITS-28S region. Sequencing was performed with an automated DNA sequencer (3730xl Genetic Analyzer; Applied Biosystems).

### Analysis of sequence data

For phylogenetic analyses, a combined matrix of ITS-LSU, SSU, *rpb2* and *tef1* sequences was produced. According to the results of GenBank nucleotide BLAST searches, the available GenBank sequences of *Curreya*, *Floricola* and *Misturatosphaeria* were aligned with the sequences of the current study. According to the tree topology of Hyde et al. (2013), GenBank sequences of selected Biatriosporaceae, Halotthiaceae, Lophiostomataceae, Melanommataceae, Pleomassariaceae, Roussoellaceae and Sporormiaceae (Table 1) were included to reveal the phylogenetic relationships of the *Teichospora* clade and to root the trees. All alignments were produced with the server version of MAFFT (www.ebi.ac.uk/Tools/mafft), checked and refined using BioEdit v.7.0.4.1 (Hall 1999). For phylogenetic analyses, all sequence alignments were combined. The final matrix contained 2072 nucleotide characters from the ITS-LSU rDNA, 2326 from the SSU rDNA, 1038 from *rpb2* and 1356 from *tef1*.

Maximum parsimony (MP) analysis of the combined matrix was performed with PAUP v.4.0a142 (Swofford 2002) using 1000 replicates of heuristic search with random addition of sequences and subsequent TBR branch swapping (MULTREES option in effect, steepest descent option not in effect). All molecular characters were unordered and given equal weight; analyses were performed with gaps treated as missing data; and the COLLAPSE command was set to minbrlen. Bootstrap analysis with 1000 replicates was performed in the same way, but using 5 rounds of random sequence addition and subsequent TBR branch swapping during each bootstrap replicate, with each replicate limited to 1 million rearrangements.

Maximum likelihood (ML) bootstrap analyses were performed with RAxML (Stamatakis 2006) as implemented in raxmlGUI 1.3 (Silvestro and Michalak 2012), using the ML+ rapid bootstrap setting and the GTRGAMMAI substitution model with 1000 bootstrap replicates. The matrix was partitioned for the individual gene regions, and substitution model parameters were calculated separately for them.

## Results

### Molecular phylogeny

**Fig. 1:**
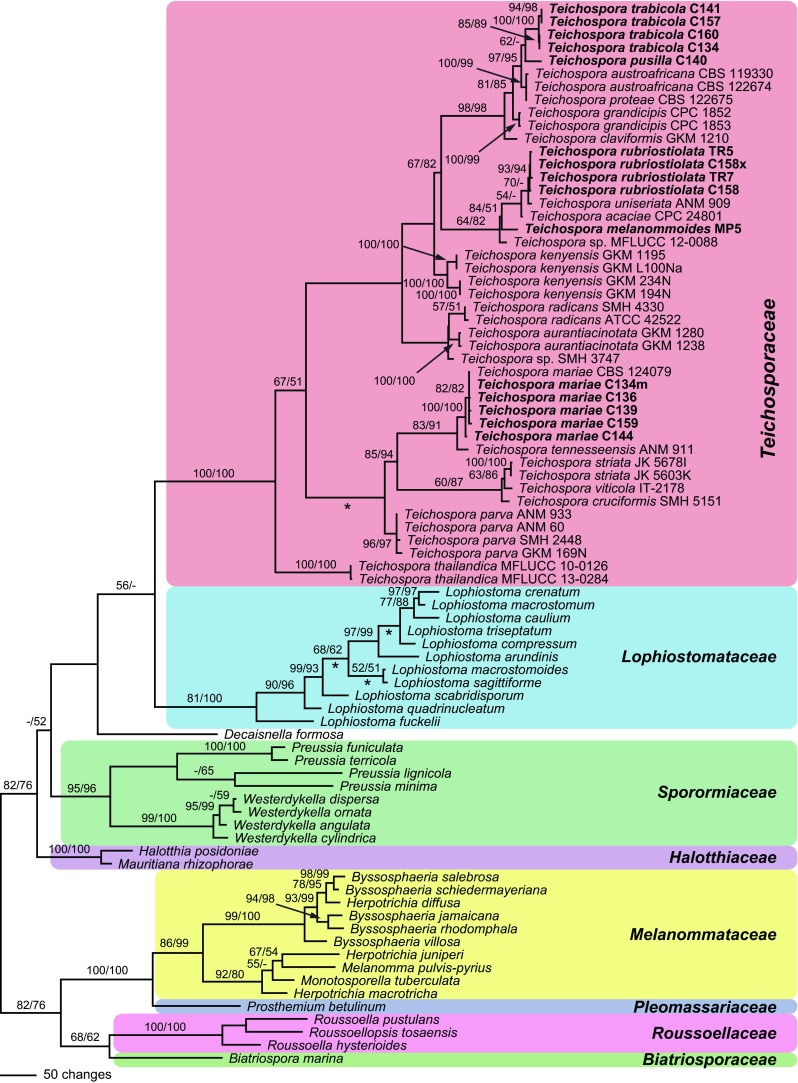
Phylogram showing 1 of 72 MP trees 5460 steps long revealed by PAUP from an analysis of the combined ITS-LSU, SSU, *rpb2* and *tef1* matrix of Teichosporaceae and related families. MP and ML bootstrap support above 50 % are given above or below the branches. For *Teichospora*, strain/culture designations are given following the taxon names. Accessions formatted in bold were sequenced in the present study. The asterisks (*) denote the nodes collapsed in the strict consensus tree of all MP trees

Of the 6792 nucleotide characters included in the phylogenetic analyses, 1323 are parsimony informative (390 of ITS-LSU, 107 of SSU, 490 of *rpb2*, 336 of *tef1*). The parsimony analyses revealed 72 MP trees of 5460 steps, one of which is shown in Fig. 1. Tree topologies of all MP trees are similar except for a few minor differences within *Lophiostoma* and a slightly different position of the *Teichospora parva* clade.

In the phylogenetic analyses, the deeper nodes mostly lack significant support, but most families are highly supported (Fig. 1). While the Teichosporaceae are highly supported, the sister group relationship to Lophiostomataceae receives only low (MP) or insignificant (ML) support. Within the Teichosporaceae, much of the backbone also lacks significant bootstrap support, but several highly supported subclades are revealed (Fig. 1). *Teichospora thailandica* is sister group to all other *Teichospora* species with moderate (MP) or low (ML) support. *Teichospora trabicola*, the generic type, is contained in a highly supported clade comprising the new species *T. pusilla*, three species formerly placed in *Curreya* (*C. austroafricana*, *C. grandicipis*, *C. proteae*) and *T.* (*Misturatosphaeria*) *claviformis*. The new species *T. melanommoides* and *T. rubriostiolata* form a moderately supported clade with *T.* (*Misturatosphaeria*) *uniseriata* and *T.* (*Curreya*) *acaciae*. Another highly supported clade contains *T. cruciformis*, *T. mariae*, *T. striata*, *T. tennesseensis* and *T. viticola*. The other species are dispersed throughout the tree without significant support.

### Taxonomy

***Teichosporaceae*** M.E. Barr, Mycotaxon 82: 374 (2002), emend.

Synonym. *Floricolaceae* Thambug., Kaz. Tanaka & K. D. Hyde, Fungal Divers. 74: 244 (2015).

Ascomata non-stromatic but sometimes surrounded or overlain by brown hyphae, immersed, erumpent to superficial, dark brown to black, perithecioid. Ostiolar necks papillate to elongate, pore rounded, interior periphysate, apex variously coloured. Peridium pseudoparenchymatous, 2–3 layered, brown. Hamathecium comprising paraphyses and pseudoparaphyses. Asci 4–8-spored, bitunicate, fissitunicate, cylindrical to subclavate. Ascospores brown, less commonly hyaline, ellipsoid, oblong, fusoid or clavate, symmetric or asymmetric, usually septate, rarely with a gelatinous sheath. Asexual morphs coelomycetous, forming pycnidia that contain brown septate or brown, rarely hyaline non-septate conidia. Saprobic in plant material.

***Teichospora*** Fuckel, Jb. nassau. Ver. Naturk. 23–24: 160 (1870) [1869–70].

= ***Floricola*** Kohlm. & Volkm.-Kohlm., Bot. Mar. 43: 385 (2000).

= ***Misturatosphaeria*** Mugambi & Huhndorf, Stud. Mycol. 64: 108 (2009).

= ***Asymmetrispora*** Thambug. & K.D. Hyde, Fungal Divers. 74: 247 (2015).

= ***Aurantiascoma*** Thambug. & K.D. Hyde, Fungal Divers. 74: 249 (2015).

= ***Magnibotryascoma*** Thambug. & K.D. Hyde, Fungal Divers. 74: 249 (2015).

= ***Neocurreya*** Thambug. & K.D. Hyde, Fungal Divers. 74: 249 (2015).

= ***Pseudoaurantiascoma*** Thambug. & K.D. Hyde, Fungal Divers. 74: 250 (2015).

= ***Pseudomisturatosphaeria*** Thambug. & K.D. Hyde, Fungal Divers. 74: 251 (2015).

= ***Ramusculicola*** Thambug. & K.D. Hyde, Fungal Divers. 74: 251 (2015).

Type species: *Teichospora trabicola* Fuckel (designated by Fuckel 1870).

Ascomata immersed, erumpent to superficial, occurring singly or aggregated in clusters, globose to pyriform, dark brown, darkening in KOH; peridium complex, sometimes surrounded by brown hyphae penetrating into the wood; ostiolar neck distinct, circular in outline, with rounded or flat, variously coloured top; ostiole periphysate. Hamathecium of numerous septate, branching, apically free paraphyses and pseudoparaphyses. Asci cylindrical to subclavate, bitunicate, fissitunicate, short stipitate, containing 4–8 uni- to partly biseriately arranged ascospores. Ascospores ellipsoid to clavate, brown, rarely hyaline, didymo-, phragmo- or dictyosporous, not or slightly constricted at the septa, usually smooth and without a sheath. Asexual morphs coelomycetous, pycnidial, with conidiophores reduced to conidiogenous cells and brown unicellular (coniothyrium-like), rarely hyaline unicellular (aposphaeria-like) or several-celled brown conidia, only known for few species. Saprobic in wood, bark or leaves.

**Fig. 2:**
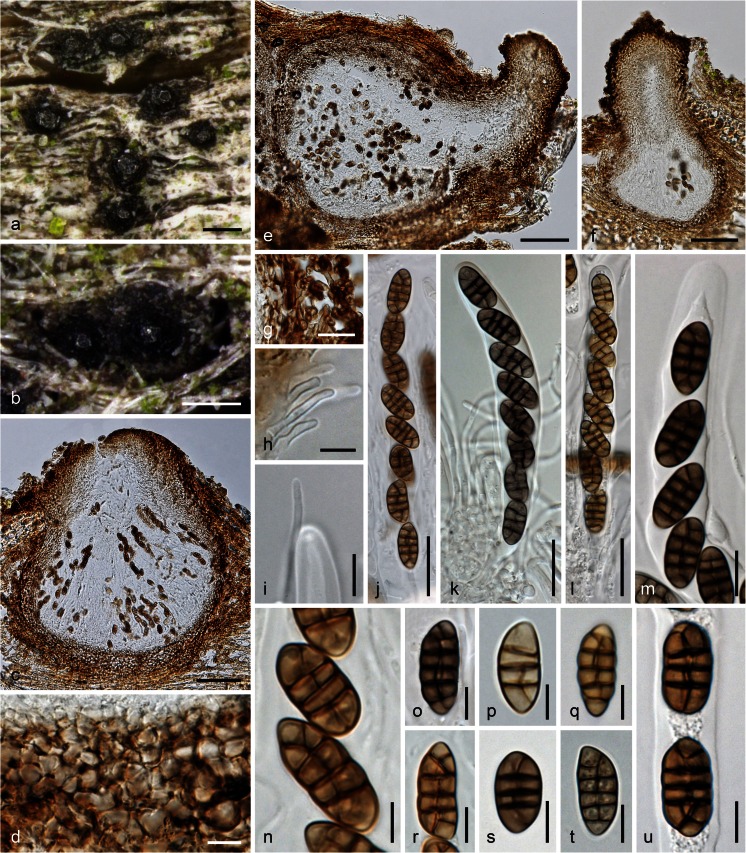
*Teichospora trabicola*. **a**, **b** Ascomata (Ostioles) in face view. **c**, **e**, **f** Ascomata in vertical section. **d** Basal ascomatal wall in section. **g** Hyphae at lateral peridium. **h** Periphyses. **i** Apically free paraphysis with tip of immature ascus. **j**–**l** Asci (**k** in 3 % KOH). **m** Ascus apex in 3 % KOH. **n**–**u** Ascospores (**o**, **s**–**u** in 3 % KOH). **a**–**h**, **l**, **m**, **p**, **q**, **s**, **u** WU 33582 (C134); **i**, **k**, **o** isolectotype NY; **j**, **n**, **r** lectotype BPI 626159; **t** WU 33584 (C157). *Scale bars* (**a**,** b**) 200 μm; (**c**,** e**,** f**) 70 μm; (**d**,** i**,** m**) 10 μm; (**g**,** j**–**l**) 20 μm; (**h**,** o**,** q**–**u**) 7 μm; (**n**,** p**) 5 μm

***Teichospora trabicola*** Fuckel, Jb. nassau. Ver. Naturk. 23–24: 161 (1870) [1869–70]. Fig. 2

Ascomata (175)230–350(397) μm diam (*n* = 29), (236)300–412(437) μm high (*n* = 21), variable in shape and size, when immersed pyriform or consisting of a small subglobose venter and a long, sometimes curved, cylindrical to conical ostiolar neck, when erumpent comprising a globose venter and a short ostiolar papilla. Peridium (30)34–61(80) μm thick (*n* = 32), consisting of a narrow, ill-defined, hyaline to pale brownish or olivaceous inner layer, becoming thicker and more distinctly cellular toward the ostiole, a dark brown *textura angularis* of mostly laterally compressed, at the base isodiametric cells of (4)5–10(13) μm diam (*n* = 50) that become darker, thicker-walled and irregularly pigmented to the outside, turning dark greyish brown in 3 % KOH; surrounded by dark brown, 2–5(6.5) μm wide hyphae fraying out and penetrating into wood, often compacted at the host surface to form a clypeus-like structure visible as a dark crust; not darkening in KOH. Ostioles (78)120–185(215) μm long, (56)60–106(141) μm wide at the apex (*n* = 17), periphysate, neck (66)90–155(205) μm wide at the apex (*n* = 32). Hamathecium of numerous apically free paraphyses and branched pseudoparaphyses 1–3 μm wide. Asci arranged in basal hymenium, (80)102–129(132) × (11)12–15(16.5) μm (*n* = 15), cylindrical, bitunicate, fissitunicate, containing 8 (obliquely) uniseriate ascospores; endotunica narrow, swelling in 3 % KOH; stipe short, base simple. Ascospores (13.5)15.0–18.5(21.8) × (5.5)7.3–8.8(10.1) μm, l/w (1.7)1.9–2.3(2.8) μm (*n* = 150), ellipsoid to oblong, symmetrical to slightly asymmetrical, with 3(–4–5) thick, non-constricted eusepta, 1 or 2 V- or Y-septa in terminal cells and a longitudinal septum in a variable number of cells; septa thicker than the wall, subhyaline to yellowish when young, turning dark brown, turning darker to nearly black in 3 % KOH, smooth, without a sheath.

Asexual morph: unknown.

Ecology: In exposed wood of *Quercus* and *Robinia pseudoacacia*.

Distribution: Europe (Austria, Germany); so far not known outside wine-growing regions.

Typification. Lectotype, here designated: Germany, Hessen, Oestrich, on oak poles in a vineyard, in winter, L. Fuckel (BPI 626159, MBT 203894; NY: isolectotype). Epitype, here designated: Austria, Niederösterreich, Gumpoldskirchen, vineyards above Melkerhof, on vineyard poles of *Robinia pseudoacacia*, soc. *T. mariae*, 9 Nov 2014, H. Voglmayr, W. Jaklitsch & I. Greilhuber (WU 33582; MBT 203895; culture CBS 140730 = C134). In the original type materials it is very difficult to find ascomata useful for morphological examination. Ascomata are mostly overmature, broken and effete, while some are immature. This is the reason for epitype designation. Hyde et al. (2013) illustrated *T. trabicola* (fig. 119 on p. 245) from the lectotype with immature asci. However, the slides prepared by R. Phookamsak for that work had not yet dried out completely and have thus been useful to also illustrate mature asci and ascospores here (see Fig. 2). The lectotype material perfectly matches that from NY, including hyphae surrounding ascomata and the peridial structure. The description above is exclusively based on the examination of type and fresh material. The *T. trabicola* type folder in G (G 00110113) does not contain the fungus, but only a black crust on pieces of bark, issued by Fuckel as *Torula antiqua* Corda, regarding it as one of two asexual morphs of *T. trabicola*. The lecto- and isolectotypes and also the part of FH 00545643 received (no ascomata found) contain also pycnidia with conidia resembling ascospores but with verrucose ornamentation and a slightly larger size (16.5–26 × 7.5–10.5 μm), resembling conidia of *Floricola* except for a vertical septum in one or a few cells.

*Other materials examined*: Austria, Niederösterreich, Enzersfeld, on vineyard poles of *Robinia pseudoacacia*, 3 Oct 2015, W. Jaklitsch (WU 33586); Gumpoldskirchen, vineyards above Melkerhof, on vineyard poles of *Robinia pseudoacacia*, 1 Mar 2015, W. Jaklitsch & H. Voglmayr (WU 33583; culture C141). Germany, Hessen, Rheingau, Oestrich, vineyards between Kühns Mühle and Oestrich forest, on vineyard poles of *Robinia pseudoacacia*, 3 Apr 2015, W. Jaklitsch (WU 33584; culture C157); same area and date, different vineyard, on vineyard poles of *Robinia pseudoacacia*, W. Jaklitsch (WU 33585; culture C160).

Notes: Fresh material perfectly matches type material. Ostiolar necks are very variable, depending on the degree of immersion in the wood. Asci are fissitunicate but remarkably stable in microscopic mounts, particularly in KOH, which is one reason for its use in microscopy. Paraphyses with free apices have been detected among young asci (also verified in the isolectotype), suggesting that they are true paraphyses.

***Teichospora acaciae*** (Crous & M.J. Wingf.) Jaklitsch & Voglmayr, comb. nov., MycoBank MB 815654

Basionym. *Curreya acaciae* Crous & M.J. Wingf., in Crous et al., Sydowia 67: 94 (2015b).

***Teichospora aurantiacinotata*** (Mugambi & Huhndorf) Jaklitsch & Voglmayr, comb. nov.

MycoBank MB 815655

Basionym. *Misturatosphaeria aurantiacinotata* Mugambi & Huhndorf [as ‘aurantonotata’], Stud. Mycol. 64: 108 (2009).

***Teichospora austroafricana*** (Marinc., M.J. Wingf. & Crous) Jaklitsch & Voglmayr, comb. nov.

MycoBank MB 815656

Basionym. *Curreya austroafricana* Marinc., M.J. Wingf. & Crous, in Marincowitz et al. CBS Diversity Ser. (Utrecht) 7: 37 (2008).

Syn. *Neocurreya austroafricana* (Marinc., M.J. Wingf. & Crous) Thambug. & K.D. Hyde, Fungal Divers. 74: 250 (2015).

***Teichospora claviformis*** (Mugambi & Huhndorf) Jaklitsch & Voglmayr, comb. nov.

MycoBank MB 815657

Basionym. *Misturatosphaeria claviformis* Mugambi & Huhndorf, Stud. Mycol. 64: 113 (2009).

Syn. *Neocurreya claviformis* (Mugambi & Huhndorf) Thambug. & K.D. Hyde, Fungal Divers. 74: 250 (2015).

***Teichospora cruciformis*** (Mugambi & Huhndorf) Jaklitsch & Voglmayr, comb. nov.

MycoBank MB 815658

Basionym. *Misturatosphaeria cruciformis* Mugambi & Huhndorf, Stud. Mycol. 64: 113 (2009).

Syn. *Pseudomisturatosphaeria cruciformis* (Mugambi & Huhndorf) Thambug. & K.D. Hyde, Fungal Divers. 74: 251 (2015).

***Teichospora grandicipis*** (Joanne E. Taylor & Crous) Jaklitsch & Voglmayr, comb. nov.

MycoBank MB 815659

Basionym: *Coniothyrium grandicipis* Joanne E. Taylor & Crous, in Crous et al. CBS Diversity Ser. (Utrecht) 2: 60 (2004).

Syns. *Curreya grandicipis* (Joanne E. Taylor & Crous) Joanne E. Taylor & Crous, in Crous et al. Persoonia 27: 32 (2011).

*Neocurreya grandicipis* (Joanne E. Taylor & Crous) Thambug. & K.D. Hyde, Fungal Divers. 74: 250 (2015).

***Teichospora kenyensis*** (Mugambi & Huhndorf) Jaklitsch & Voglmayr, comb. nov.

MycoBank MB 815660

Basionym. *Misturatosphaeria kenyensis* Mugambi & Huhndorf, Stud. Mycol. 64: 113 (2009).

Syn. *Pseudoaurantiascoma kenyense* (Mugambi & Huhndorf) Thambug. & K.D. Hyde, Fungal Divers. 74: 250 (2015).

Notes: Mugambi and Huhndorf (2009) listed only the holotype (GKM1195) in their description, two collections (GKM1195 and GKM L100Na) are given in their figure legends, but sequences of four collections from at least two different localities were included in their molecular phylogenies. In their phylogenetic tree, two distinct highly supported subclades are revealed within *T. kenyensis*, with genetic differences comparable to those between distinct species in other *Teichospora* lineages; e.g. between T. *mariae* and *T. tennesseensis* (Fig. 1). Therefore, *T. kenyensis* may actually comprise two closely related species.

***Teichospora mariae*** (Ying Zhang, J. Fourn. & K.D. Hyde) Jaklitsch & Voglmayr, comb. nov.

**Fig. 3:**
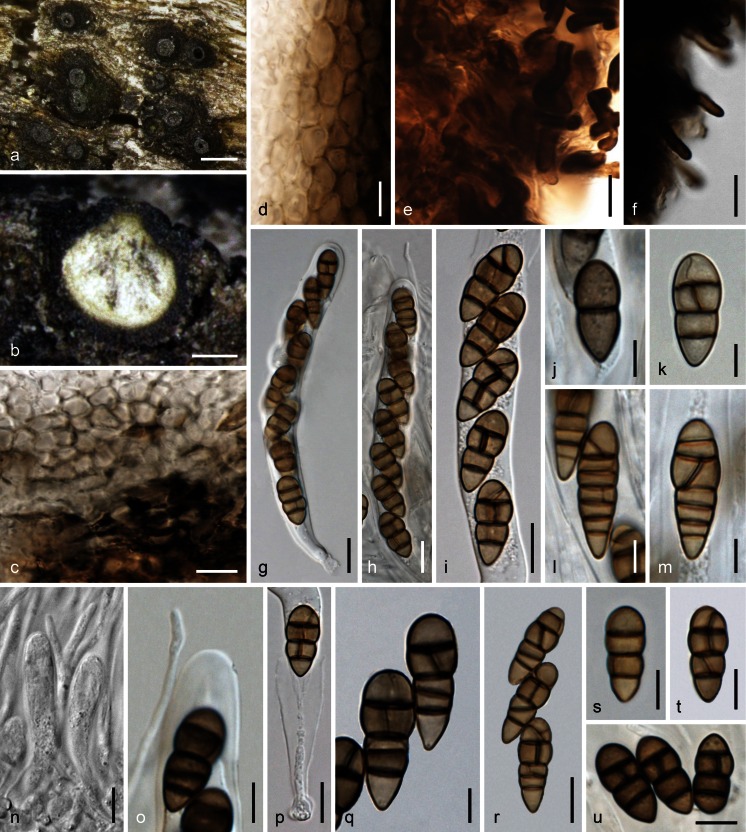
*Teichospora mariae*. **a** Ascomata (Ostioles) in face view. **b** Ascoma in vertical section. **c** Basal ascomatal wall in section. **d** Lateral ascomatal wall in section. **e** Hyphae at lateral peridium near the base. **f** Setae at lower ascomatal side. **g**, **h** Asci. **i**–**m**, **q**–**u** Ascospores (**j**, **q**, **u** in 3 % KOH). **n** Apically free paraphyses among young asci. **o** Ascus apex and paraphysis tip in 3 % KOH. **p** Ascus base (showing fissitunicate dehiscense). **a**, **b**, **k**, **q** WU 33587 (C140m); **c**–**f**, **n** WU 33588 (C136); **g**, **h**, **j**, **o**, **s**, **u** WU 33590 (C144); **i**, **l**, **m**, **p**, **r**, **t** WU 33592 (C134m). *Scale bars* (**a**) 200 μm; (**b**) 100 μm; (**c**,** e**,** g**–**i**,** p**,** r**) 10 μm; (**d**,** f**,** n**,** s**–**u**) 7 μm; (**j**–**m**,** o**,** q**) 5 μm

MycoBank MB 815661. Fig. 3.

Basionym. *Misturatosphaeria mariae* Ying Zhang, J. Fourn. & K.D. Hyde, Mycoscience 54: 2 (2012) [2013].

Syn. *Asymmetrispora mariae* (Ying Zhang, J. Fourn. & K.D. Hyde) Thambug. & K.D. Hyde, Fungal Divers. 74: 248 (2015).

Ascomata scattered or aggregated in small groups, immersed to erumpent with the lower third remaining immersed, subglobose to pyriform, 230–530 μm diam, 300–400 μm high; ostiolar necks distinct, 70–150 μm long, 70–142 μm wide (*n* = 15) at the apex, cylindrical to broadly conical, elongated when deeply immersed or reduced to a papilla when nearly superficial; top often flattened discoid and shiny black, rarely orange brown; ostiole periphysate. Peridium 30–45 μm thick at base and sides, comprising a hyaline to olivaceous inner layer of isodiametric and thin-walled, 4–8 μm wide cells, becoming thicker toward the ostiole, and a dark brown outer layer of slightly larger, up to 11.5 μm long, compressed, thick-walled cells; surrounded by dark brown, (1.5)2.5–5.7 μm wide hyphae and short terminally narrowly rounded, 1.5–3 μm wide setae at lower parts of the ascoma. Hamathecium consisting of numerous septate, apically free paraphyses and branched pseudoparaphyses, (1)1.5–3 μm wide. Asci arranged in a basal hymenium, (78)91–114(126) × (11)11.5–14(15.5) μm (*n* = 12), cylindrical to oblong, rarely subclavate, bitunicate, fissitunicate, with a short stipe and simple or knob-like base, containing 8 (obliquely) uniseriate, partly biseriate or overlapping ascospores; walls narrow, endotunica swelling in 3 % KOH. Ascospores (13)15.5–19.7(21.5) × (6.2)6.8–8(9) μm, l/w (1.9)2.1–2.7(3.3) (*n* = 60), ovoid to clavate, lower end rounded or acute, straight, first hyaline, 1–2-celled, becoming pale to dark brown, darkening in 3 % KOH, with 3(–4–6) thick and dark transverse septa and 1 longitudinal or oblique septum in 1–3 cells and sometimes a V- or Y-septum in the upper end cell, constricted at the more or less median primary septum, smooth, without a sheath.

Asexual morph: unknown.

Ecology: In bark and wood of *Robinia pseudoacacia*.

Distribution: Europe (Austria, France, Germany).

*Materials examined*: Austria, Niederösterreich, Gumpoldskirchen, vineyards above Melkerhof, on standing vineyard poles of *Robinia pseudoacacia*, soc. *T. trabicola*, 9 Nov 2014, H. Voglmayr, W. Jaklitsch & I. Greilhuber (WU 33592; culture CBS 140732 = C134m); same area, different vineyard, on vineyard poles of *Robinia pseudoacacia*, soc. *T. pusilla*, 1 Mar 2015, W. Jaklitsch & H. Voglmayr (culture C140m); Wagram, Hippersdorf, on vineyard poles of *Robinia pseudoacacia*, 15 Nov 2014, W. Jaklitsch (WU 33588; culture C136); Mühlleiten, on old bark of standing *Robinia pseudoacacia*, 28 Feb 2015, H. Voglmayr (WU 33589; culture C139). Vienna, 22nd district, Lobau, near Panozzalacke, on old bark of standing *Robinia pseudoacacia*, soc. *Strickeria kochii*, 7 Mar 2015, W. Jaklitsch (WU 33590; culture C144). France, Ariège, Rimont, Las Muros, elev. ca. 470 m, on dead hanging branch of *Robinia pseudoacacia*, on decorticated rotten sapwood, ostiolar pore area orange brown, 22 May 2015, J. Fournier JF 15050. Germany, Hessen, Rheingau, Oestrich, vineyards between Kühns Mühle and Oestrich forest, on vineyard poles of *Robinia pseudoacacia*, 3 Apr 2015, W. Jaklitsch (WU 33591; culture C159).

Notes: *Teichospora mariae*, originally described by Zhang et al. (2013), is here redescribed in order to emend the morphological description. This species is common on *Robinia*, often in association with other species of *Teichospora* such as *T. pusilla*, *T. trabicola* or *T. rubriostiolata*, and also with *Strickeria kochii*. Ascomata are indistinguishable from those of *T. trabicola*, but the ovoid to clavate ascospores and the ascomatal setae set it apart from this species. Furthermore, it not only occurs on vineyard poles but also in wood and bark of branches and standing trunks of *Robinia*. Remarkably, it is so far unknown from North America, the origin of its host.

*Teichospora brevirostris* (Fr.) Fuckel, which was based on *Sphaeria brevirostris* Fr. 1823, was evaluated as a putative older name for *T. mariae*. A reason for this is the description and illustration of the only species with clearly ovoid to clavate ascospores of similar size and non-collabent ascomata by Berlese (1900, p. 50, pl. LXXI, fig. 2). There is a single specimen of *Sphaeria brevirostris* in UPS, which was distributed as Scleromyceti Sueciae, ed. 2, no. 390 (F-118034) from 1834 on (Holm and Nannfeldt 1962). Therefore, this undated specimen is not a type specimen. Examination of this specimen showed perithecia erumpent from old wood, but no ascospores were found. Another part of this specimen from editio 2 (or editio nova), present in G (G 00111994), contains two different species, a scolecosporous pyrenomycete and another pyronemycete with larger ascomata only containing a few brown phragmospores. Another specimen in G (G 00111995), received by Fuckel from Rabenhorst, contains *Clypeosphaeria mamillana*. In effect, *Sphaeria brevirostris* cannot be interpreted on a sound basis and should be considered a nomen dubium. Fuckel (1870) described *T. brevirostris* from pine wood and Berlese (1900) presented material of Saccardo from *Quercus*, corroborating Fuckel’s concept of the species. Although ascospore shape and size may be similar, ascospores of this species are described as 5-septate muriform by Fuckel, and the ascospore septation shown by Berlese (1900) does not perfectly match *T. mariae*, which has ascospores with chiefly 3(–4) septa. The different hosts may be further evidence for non-conspecificity, as *T. mariae* is so far only known from *Robinia pseudoacacia* on which it is common.

***Teichospora melanommoides*** Jaklitsch, Friebes & Voglmayr, sp. nov.

**Fig. 4:**
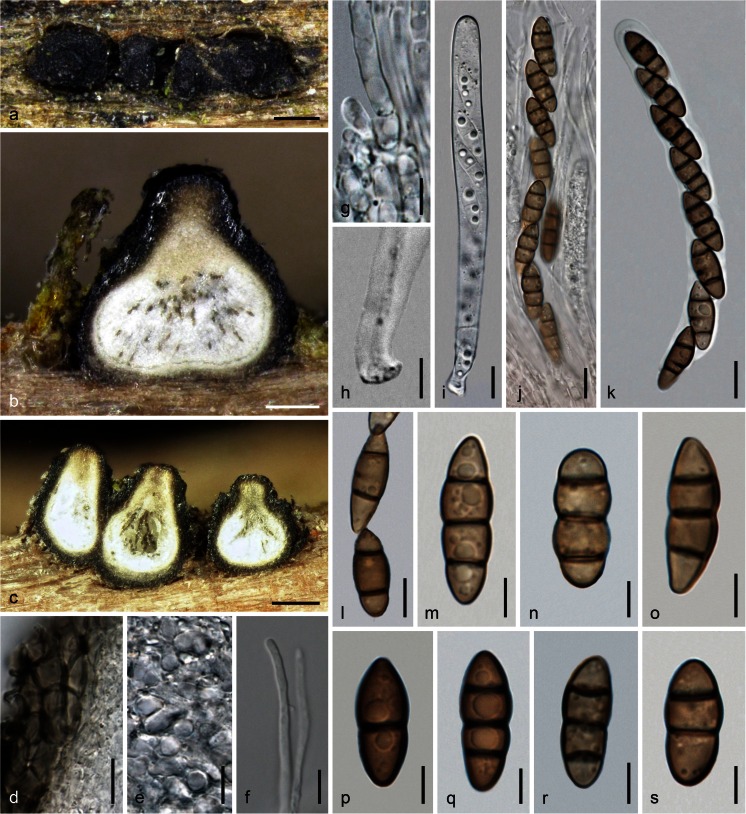
*Teichospora melanommoides* (WU 33593).** a** Ascomata in face view.** b**,** c** Ascomata in vertical section.** d** Lateral ascomatal wall in section.** e** Inner hyaline part of peridium near ostiole.** f** Apically free paraphysis.** g** Basal part of paraphyses.** h** Ascus base.** i**–**k** Asci (**i** immature;** k** in 3 % KOH).** l**–**s** Ascospores (**m**,** p**–**s** in 3 % KOH). *Scale bars* (**a**) 300 μm; (**b**) 100 μm; (**c**) 150 μm; (**d**,** i**–**k**) 10 μm; (**e**,** f**,** l**) 7 μm; (**g**,** h**,** m**–**s**) 5 μm

MycoBank MB 815662. Fig. 4.

Etymology: *melanommoides*, referring to its similarity with species of *Melanomma*.

Ascomata scattered or aggregated in groups of 2–4, erumpent-superficial on wood, subglobose or pyriform, (265)326–486(580) μm high, (283)323–500(585) μm diam (*n* = 29). Peridium 30–60 μm thick, consisting of a narrow hyaline inner layer becoming thicker and more distinctly cellular toward the ostiole, and a brown outer *t. angularis*, of thin-walled, (2.5)4.3–9.0(11) μm wide (*n* = 20) cells, becoming darker and thick-walled towards the outside, turning nearly black, partly olivaceous in 3 % KOH; surface with some protruding cells near the ostiole and surrounded by scant brown, ca. 2–5 μm wide hyphae at the base. Ostiolar necks (88)114–192(212) μm diam (*n* = 20), stout, often shiny black, papillate to broadly conical with rounded or flattened top of circular outline; ostiole periphysate. Hamathecium consisting of numerous, (1)1.5–3.5(4) μm wide, apically free paraphyses and pseudoparaphyses, septate, branched; basal part submoniliform. Asci (107)113–135(138) × (10.3)10.5–12.5(13.7) μm (*n* = 8), cylindrical, bitunicate, fissitunicate, with a short stipe and a knob-like base, narrow walls with endotunica swelling in 3 % KOH; containing 6–8 ascospores in (obliquely) uniseriate to partly biseriate arrangement in upper region, unstable in water. Ascospores (14.5)16.0–19.8(22.3) × (5.5)6.2–7.5(8.2) μm, l/w (2)2.3–2.9(3.2) (*n* = 50), narrowly ellipsoid, oblong to fusoid, straight, with 1–3 eusepta, slightly constricted at all septa, medium to dark brown, darkening in 3 % KOH, end cells often conical and sometimes lighter, multiguttulate, smooth, without a sheath.

Ecology: In wood of *Salix*.

Distribution: Only known from the holotype.

Holotype: Austria, Steiermark, Bruck an der Mur, nature reserve between Pichl-Großdorf and Tragöß-Oberort, on a decorticated branch of *Salix* sp., soc. coniothyrium-like coelomycete, 25 May 2015, G. Friebes (WU 33593; culture CBS 140733 = MP5).

Notes: Based on morphology alone, *Teichospora melanommoides* would be determined as a species of *Melanomma*. The holotype specimen contains many ascomata, but most are overmature or effete; therefore, it was difficult to find intact asci for illustration, and the ascomata were not sectioned.

***Teichospora parva*** Jaklitsch & Voglmayr, nom. nov.

MycoBank MB 815663

Etymology: parva, referring to the small ascomata.

Replaced synonym. *Misturatosphaeria minima* Mugambi, A.N. Mill. & Huhndorf, in Mugambi & Huhndorf, Stud. Mycol. 64: 114 (2009), non *Teichospora minima* Ellis & Everh., Proc. Acad. nat. Sci. Philad. 47: 419 (1895).

Syn. *Aurantiascoma minimum* (Mugambi, A.N. Mill. & Huhndorf) Thambug. & K.D. Hyde, Fungal Divers. 74: 249 (2015).

Notes: This species was differentiated from others in Mugambi and Huhndorf (2009) by smaller ascomata. As the epithet *minima* is already occupied by *Teichospora minima* Ellis & Everh., a new epithet was necessary.

***Teichospora proteae*** (Marinc., M.J. Wingf. & Crous) Jaklitsch & Voglmayr, comb. nov.

MycoBank MB 815664

Basionym. *Curreya proteae* Marinc., M.J. Wingf. & Crous, in Marincowitz et al. CBS Diversity Ser. (Utrecht) 7: 39 (2008).

Syn. *Neocurreya proteae* (Marinc., M.J. Wingf. & Crous) Thambug. & K.D. Hyde, Fungal Divers. 74: 250 (2015).

Note: *Teichospora* (*Curreya*) *proteae* was differentiated in Marincowitz et al. (2008) by larger ascopore size ((17)18–19(22.5) × (7)8–9(11) μm) from *T. austroafricana* ((14)14.5–16(17) × (6)7–8(9) μm). As the ITS and LSU ex-type sequences of both species are identical, additional markers with higher resolution should be sequenced to evaluate their status as distinct species.

***Teichospora pusilla*** Jaklitsch & Voglmayr, sp. nov.

**Fig. 5:**
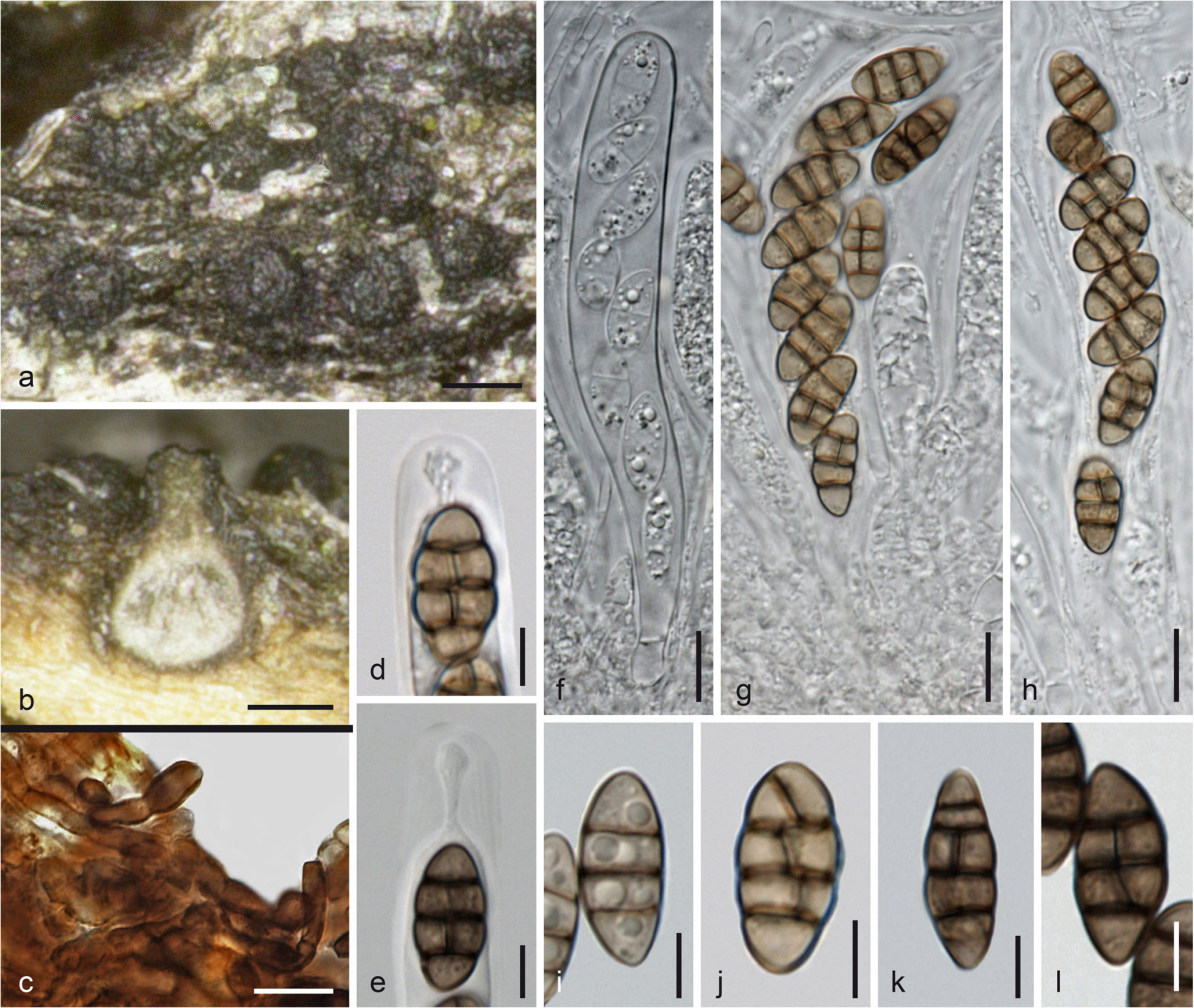
*Teichospora pusilla* (WU 33587). **a** Pulvinate ostioles in face view. **b** Ascoma in vertical section. **c** Hyphae at lateral peridium. **d**, **e** Ascus apices (**e** in 3 % KOH). **f**–**h** Asci (**f** immature). **i**–**l** Ascospores (**i** young; **i**, **k**, **l** in 3 % KOH). *Scale bars* (**a**,** b**) 100 μm; (**c**,** f**–**h**) 10 μm; (**d**,** e**,** i**–**l**) 5 μm

MycoBank MB 815665. Fig. 5.

Etymology: *pusilla*, referring to its small ascomata and ascospores.

Ascomata scattered or aggregated in small groups, immersed in wood, globose to pyriform, (230)240–310(336) μm high, (140)155–320(372) μm diam (*n* = 10). Peridium ca. 13–30 μm thick, consisting of a narrow hyaline inner *t. angularis* of thin-walled isodiametric cells and an outer *t. angularis* of ca. 4–14 μm wide, dark brown, thick-walled cells, turning dark greyish brown in 3 % KOH; surrounded by dark brown, 2–5 wide hyphae. Ostiolar necks distinct, papillate or short-cylindrical, apex (62)75–110(125) μm diam (*n* = 20), rounded, appearing pulvinate on the host surface, pierced by a minute central pore. Ostioles periphysate. Hamathecium of numerous, 1.0–3.5(4.5) μm wide, apically free paraphyses and pseudoparaphyses, septate, branched. Asci (86)88–94(95) × (11)11.2–14.5(15.5) μm (*n* = 5), cylindrical to subclavate, bitunicate, fissitunicate, with a short stipe and simple or knob-like base, containing 8 obliquely uniseriate ascospores. Ascospores (11.7)13.0–15.0(16.2) × (5.7)6.3–7.5(8) μm, l/w (1.6)1.9–2.2(2.6) (*n* = 33), (mostly broadly) ellipsoid, straight, with 3 slightly constricted eusepta and 1 longitudinal septum in 1–3 cells, medium to dark brown, darkening in 3 % KOH, smooth, without a sheath.

Ecology: In wood of vineyard poles.

Distribution: Only known from the holotype.

Holotype: Austria, Niederösterreich, Gumpoldskirchen, vineyards above Melkerhof, on vineyard poles of *Robinia pseudoacacia*, soc. *T. mariae*, 1 Mar 2015, W. Jaklitsch & H. Voglmayr (WU 33587; culture CBS 140731 = C140).

Notes: Ostiolar necks, ascomata, asci and ascospores of *Teichospora pusilla* are considerably smaller than in *T. trabicola*. However, ascospores are similar in both species, but in *T. pusilla* ascospores have shorter end cells and thinner transverse septa, which are more regularly and more distantly inserted. Unlike *T. trabicola*, ascospores of *T. pusilla* are also slightly constricted at the septa, and V- or Y-septa are uncommon. The holotype specimen contains this fungus in small ascomatal numbers but *T. mariae* in excess, therefore the material was not sectioned in order to save material.

***Teichospora radicans*** (Samuels & E. Müll.) Jaklitsch & Voglmayr, comb. nov.

MycoBank MB 815666

Basionym. *Melanomma radicans* Samuels & E. Müll., Sydowia 31: 147 (1979) [1978].

Syns. *Misturatosphaeria radicans* (Samuels & E. Müll.) Thambug. & K.D. Hyde, Fungal Divers. 74: 247 (2015).

*Misturatosphaeria uniseptata* Mugambi, A.N. Mill. & Huhndorf, in Mugambi & Huhndorf, Stud. Mycol. 64: 114 (2009).

***Teichospora rubriostiolata*** Jaklitsch & Voglmayr, sp. nov.

**Fig. 6:**
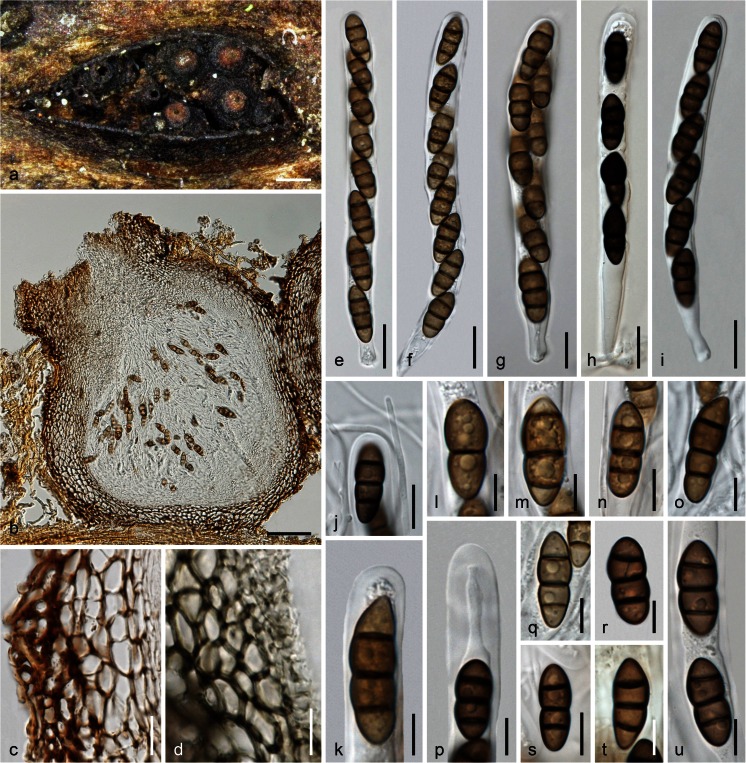
*Teichospora rubriostiolata*. **a** Ascomata (Ostioles) in face view. **b** Ascoma in vertical section. **c**, **d** Lateral ascomatal wall in section (**c** in lactic acid; **d** in 3 % KOH). **e**–**i** Asci (**h**,** i** in 3 % KOH). **j**, **k**, **p**. Ascus apices (**j**, **p** in 3 % KOH. **j** showing free paraphysis tip;** p** showing swollen endotunica). **l**–**o**, **q**–**u** Ascospores (**r**–**u** in 3 % KOH). **a**, **f**, **h**, **k**–**n**, **s**, **t** WU 33595 (TR5); **b**–**e**, **g**, **i**, **j**, **o**, **p**, **r**, **u** WU 33594 (TR7); **q** WU 33596 (C158). *Scale bars* (**a**) 200 μm; (**b**) 50 μm; (**c**,** d**,** g**,** j**,** n**,** s**) 10 μm; (**e**,** f**,** h**,** i**) 15 μm; (**k**–**m**,** o**–**r**,** t**,** u**) 7 μm

MycoBank MB 815667. Fig. 6.

Etymology: *rubriostiolata*, referring to the reddish ostiolar discs.

Ascomata scattered or aggregated in groups to ca. 40, erumpent from well-decayed, sometimes reddish pigmented bark or immersed-erumpent-superficial on wood, globose, subglobose or pyriform, (300)373–450(480) μm high, (218)290–430(515) μm diam (*n* = 45). Peridium (33)37–52(60) μm thick (*n* = 34), consisting of a narrow hyaline, non-descript inner layer of small and thin-walled cells tending to be thicker toward the ostiole and a pigmented outer *t. angularis* with cells (5)7.5–12(14.5) × (3.7)4.7–8.0(9.3) μm (*n* = 20), with walls becoming thicker toward the outside, reddish brown in lactic acid and glycerol, nearly black in 3 % KOH; brown, 2–5 μm wide hyphae present around ascomata at their bases, scant. Ostiolar necks distinct, papillate or short-cylindrical, apex (88)127–202(250) μm (*n* = 50) wide, flattened discoid, reddish-orange or black, sometimes rounded. Ostioles (98)109–164(184) μm long, apically (43)61–114(140) μm wide (*n* = 17), periphysate. Hamathecium of numerous, (1)1.5–2.5(3.5) μm wide, apically free paraphyses and pseudoparaphyses, septate, branched. Asci (80)97–128(148) × (10)10.3–13.5(17.5) μm (*n* = 23), cylindrical or oblong, bitunicate, fissitunicate, with a short stipe and simple or knob-like base, walls narrow, endotunica swelling in KOH; containing (4)6–8 uniseriate, less commonly partly biseriate ascospores. Ascospores (14)17–21(25) × (5.3)6.5–8.3(9.5) μm, l/w (2.1)2.3–2.9(3.5) (*n* = 69), narrowly ellipsoid, oblong to subfusoid, often slightly attenuated downward, straight to slightly curved, with (1)3 eusepta, strongly constricted at the median primary septum, not or only slightly at other septa, first hyaline to pale brown, turning dark brown when mature, darkening in 3 % KOH, end cells sometimes lighter, smooth, without a sheath.

Ecology: In bark and wood of trees and shrubs including vineyard poles (*Ribes*, *Robinia*, *Vaccinium*).

Distribution: Europe (Belgium, Germany, Norway).

Holotype: Norway, Aust-Agder, Grimstad kommune, Dǿmmersmoen, on twigs of *Ribes sanguinea*, soc. coniothyrium-like coelomycete, 5 Oct 2014, W. Jaklitsch & H. Voglmayr (WU 33594; ex-type culture CBS 140734 = TR7).

*Other materials examined*: Belgium, Sint-Huibrechts-Lille, Neerpelt, on twigs of *Vaccinium myrtillus*, 6 Feb 2014, P. Bormans PB2014010 (WU 33595; culture TR5). Germany, Hessen, Rheingau, Oestrich, vineyards between Kühns Mühle and Oestrich forest, on vineyard poles of *Robinia pseudoacacia*, soc. *T. mariae*, 3 Apr 2015, W. Jaklitsch (WU 33596; culture C158 from material with black ostioles, culture C158x from material with reddish ostioles).

Notes: Specimens of *Teichospora rubriostiolata* had been identified as *Thyridaria rubronotata*. The latter differs in the presence of red to brown hyphae between ascomata, but this may vary (Chesters 1938; Wehmeyer 1941). Sometimes ostiolar apices of *T. rubriostiolata* may be entirely black. See more information about this and other issues regarding identification using morphology in the discussion.

***Teichospora striata*** (Kohlm. & Volkm.-Kohlm.) Jaklitsch & Voglmayr, comb. nov.

MycoBank MB 815668

Basionym. *Floricola striata* Kohlm. & Volkm.-Kohlm., Bot. Mar. 43: 385 (2000).

***Teichospora tennesseensis*** (Mugambi, A.N. Mill. & Huhndorf) Jaklitsch & Voglmayr, comb. nov.

MycoBank MB 815669

Basionym. *Misturatosphaeria tennesseensis* Mugambi, A.N. Mill. & Huhndorf, in Mugambi & Huhndorf, Stud. Mycol. 64: 114 (2009).

Syn. *Asymmetrispora tennesseensis* (Mugambi, A.N. Mill. & Huhndorf) Thambug. & K.D. Hyde, Fungal Divers. 74: 248 (2015).

***Teichospora thailandica*** (Thambug. & K.D. Hyde) Jaklitsch & Voglmayr, comb. nov.

MycoBank MB 815670

Basionym. *Ramusculicola thailandica* Thambug. & K.D. Hyde, Fungal Divers. 74: 251 (2015).

***Teichospora uniseriata*** (Mugambi, A.N. Mill. & Huhndorf) Jaklitsch & Voglmayr, comb. nov.

MycoBank MB 815671

Basionym. *Misturatosphaeria uniseriata* Mugambi, A.N. Mill. & Huhndorf, in Mugambi & Huhndorf, Stud. Mycol. 64: 116 (2009).

Syn. *Magnibotryascoma uniseriatum* (Mugambi, A.N. Mill. & Huhndorf) Thambug. & K.D. Hyde, Fungal Divers. 74: 249 (2015).

***Teichospora viticola*** (Phukhams., Camporesi & K.D. Hyde) Jaklitsch & Voglmayr, comb. nov.

MycoBank MB 815672

Basionym. *Floricola viticola* Phukhams., Camporesi & K.D. Hyde, in Ariyawansa et al., Fungal Divers. 75: 71 (2015).

### Other species

The original account of *Teichospora* listed the five species *Teichospora brevirostris*, *T. dura*, *T. morthieri*, *T. obducens* and *T. trabicola*. For *T. brevirostris*, see the notes under *T. mariae. Teichospora dura* is the generic type of *Teichosporella*, characterised by discoid ascomata, a disaggregating upper peridium and hyaline dictyospores in clavate asci. It was assigned to the Patellariales by Barr (1981), which was refuted by Kutorga and Hawksworth (1997). No DNA data are available. *Teichospora obducens* (Schumach. : Fr.) Fuckel was erroneously treated as the generic type of *Teichospora* by von Arx and Müller (1975), but had been combined in *Cucurbitaria* by Petrak (1927), which was accepted by Barr (1990). Phylogenetically, this species neither belongs to *Teichospora* nor to *Cucurbitaria* (unpublished data). *Teichospora morthieri* was described from *Lonicera* near Neuchâtel, Switzerland. It has relatively large ascospores (32 × 11 μm) with seven transverse septa and has not been recollected recently.

Numerous other species may belong to *Teichospora*. However, DNA data are required to determine their affinities.

We have sequenced numerous fresh isolates and observed (data not shown) that many fungi of similar morphology cluster in several different families of Pleosporales. As an example, a morphologically conceived species of *Melanomma* or *Thyridaria* (having brown phragmospores) may be a member of these genera, but also of, e.g., *Teichospora*. This becomes evident only after sequencing. Therefore, it is difficult to determine which of the old names may be suitable for taxa in *Teichospora*. The use of old epithets will only be possible by collecting from the original hosts at the original places.

## Discussion

The phylogenetic position of *Teichospora* based on its type species *T. trabicola* is here clarified. This was possible, because Fuckel, who typified his genus with this species, clearly and unequivocally indicated the habitat of *T. trabicola* as occurring on vineyard poles in the winter. Collecting in his region around Oestrich at the river Rhine in the month of April yielded this species on vineyard poles, which are made of the wood of *Robinia*. These specimens contain mostly overmature ascomata, which is in line with Fuckel’s observations that good material should be searched for in winter. Furthermore, the host species is obviously less important than the ecological parameters like exposure of old decorticated wood to a rather warm and dry climate as prevails in vineyards.

NCBI Blast searches using ITS and LSU sequences of *T. trabicola* suggested a close relationship with species of *Misturatosphaeria* and recently described species of *Curreya*, and subsequent phylogenetic analyses corroborated congenericity of these species with *T. trabicola. Curreya* is younger than *Teichospora*, and its generic type, *Curreya conorum*, has not been sequenced. More has been published recently on *Misturatosphaeria*, and synonymisation of *Misturatosphaeria* under *Macrodiplodiopsis* by Wijayawardene et al. (2014) was in error. In that work. *Macrodiplodiopsis* was nicely illustrated, but the chain of activities between specimen and DNA data is obviously inconsistent. This error was corrected by Crous et al. (2015a) and acknowledged by Thambugala et al. (2015) by retaining *Misturatosphaeria* in their family Floricolaceae, a synonym of Teichosporaceae.

As outlined by Mugambi and Huhndorf (2009), the generic description of *Misturatosphaeria*, a later synonym of *Teichospora*, is broad from a morphological perspective, particularly regarding ascospores, which may be brown, sometimes hyaline, 1- to several septate or muriform. It has therefore become impossible to place pleosporalean fungi in this genus using morphology alone. We concur with Mugambi and Huhndorf (2009) concerning the scope of the genus based on molecular phylogeny, but their generic name here becomes a synonym of *Teichospora*. However, presently, we do not concur with the conclusions of Thambugala et al. (2015). They described the new species *Ramusculicola thailandica*, which is affiliated with *Teichospora*. They erected the new family Floricolaceae and produced seven new micro-genera encompassing a single or few species. Their attempt to define segregate genera having narrowly defined morphology, such as, e.g., hyaline didymospores or asymmetric brown dictyospores, may at first sight seem attractive for those who want to identify fungal species and genera by morphology alone, as ascospore colour, shape and septation are easy to determine. At second sight, however, this only pretends to the feasibility of morphological identification, which simply does not work, unfortunately, as we are inclined to say. By their procedure, the situation is in no way improved because genera with similar morphology as assembled in this family occur in several other families of Pleosporales, and therefore morphological identification is still impossible. We here discuss the morphological and phylogenetic traits and arguments.

### Evaluation of morphological characters

We generally found high intraspecific variability of several morphological traits in *Teichospora*, often even within a single specimen. Thus, several morphological characters can at best only be used for distinction at the species level, as Mugambi and Huhndorf (2009) have done. Thambugala et al. (2015), who used morphological traits for distinction at the generic level, based most of their interpretations on the descriptions by Mugambi and Huhndorf (2009) and Marincowitz et al. (2008). Mugambi and Huhndorf (2009) had based each species of *Misturatosphaeria* on a single specimen, with the exception of *M. aurantiacinotata* (two specimens), *M. minima* (four specimens), and *M. kenyensis* (morphologically on one, phylogenetically on four specimens). This may even be insufficient to estimate morphological variation at the species level.

Here, we briefly comment on some characters used by Thambugala et al. (2015) in their schematic key to genera and give some additional information and comments on other characters:

1. Numbers of ascomata: Thambugala et al. (2015) used “Ascomata solitary, or aggregated in large clusters on the host surface” versus “Ascomata solitary, or aggregated in small clusters on the host surface” for the distinction of *Misturatosphaeria* plus *Magnibotryascoma* from *Asymmetrispora* plus *Pseudomisturatosphaeria*. This cannot even be used at the species level, because such observations are simply based on the number of specimens and the method of collecting, i.e. the quality of the available specimens.
2. Position of ascomata relative to the host surface: Although Mugambi and Huhndorf (2009) described the generic type of *Misturatosphaeria*, *M. aurantiacinotata*, which they based on two specimens, with superficial ascomata, their fig. 25A also shows half-immersed, i.e. erumpent, ascomata. Our new species *Teichospora rubriostiolata* would fall phylogenetically into the “*Magnibotryascoma*” clade characterised by erumpent ascomata, but it has (erumpent to) superficial ascomata. In our experience, the position of ascomata may vary from immersed to superficial within a single specimen, in virtually all the species we studied, but also in many other ascomycetes. In this group or family, ascomata usually start off as being immersed and either remain immersed or become erumpent to superficial.
3. The peridium (ascomatal wall): According to Barr (2002), the peridium in the Teichosporaceae is 3-layered; Thambugala et al. (2015) describe the peridium of the Floricolaceae as 2-layered. Both views have their merits. The outer, pigmented layer may be variably surrounded by brown hyphae or apically rounded setae. It consists of thick-walled dark brown compressed cells, which grade into lighter brown, thinner-walled and more isodiametric cells; the latter part corresponds to the middle layer of Barr (2002). This layer may be thin or even lacking in some species. The inner hyaline layer may be lacking at lower levels of the ascomata, but is usually present and distinctly thickened at the (near) ostiolar level.
4. Ostiolar necks: Their length (long necks used to characterise *Neocurreya* by Thambugala et al. 2015) varies among ascomata even in a single specimen of a species, depending on the degree of immersion in the substrate; see Fig. 2 for *T. trabicola*, which is phylogenetically embedded within their *Neocurreya. Curreya grandicipis* is only known from its coniothyrium-like asexual morphs, and thus cannot be keyed out with long ascomatal necks.
5. Pigmentation of ostiolar apices: Although we are using this trait for an epithet, the pigmentation of the ostiolar apex may be entirely black. In the specimen WU 33596 of *Teichospora rubriostiolata* collected from vineyard poles in Fuckel’s area, only very few ascomata have an orange-reddish ostiolar top. If these were not harvested with the bulk of ascomata, the entire specimen would only consist of ascomata with black ostioles, making identification based on this character impossible. We isolated and sequenced from both forms and received identical sequences. The same situation was reported by Mugambi and Huhndorf (2009) for *Misturatosphaeria minima*: “occasionally the pore area appear orange in colour or the colouring is lacking”. This species is the type and only species of the genus *Aurantiascoma*, and “Ostiolar area orange” was used to address it in their key. We recognise it here under the new name *Teichospora parva*, because the epithet *minima* is occupied in *Teichospora*.
  Orange to reddish apices occur in species of several other genera of the Pleosporales, e.g. *Byssosphaeria*, *Chaetoplea* sensu Barr (1990), *Karstenula*, or *Thyridaria*. None of them is defined at the generic level by this morphological trait. Apart from *Teichospora rubriostiolata*, we have several other specimens which can be superficially identified as *Thyridaria rubronotata*, but molecular phylogeny places them in several unrelated families of the Pleosporales (unpublished data).
6. Hamathecium: Apparently none of the earlier authors (Barr 2002; Mugambi and Huhndorf 2009; Thambugala et al. 2015) have examined hamathecial threads in detail. It may be the doctrine that no true paraphyses occur in non-lichenized perithecioid Pleosporales (Barr 1987; Eriksson 1981) which commonly prevents the study of the hamathecium. However, in all the species of *Teichospora* we have studied, apically free paraphyses occur with immature asci, i.e. they are there from the beginning rather than being formed after detachment of pseudoparaphyses. This may be significant at the generic level, but we have not seen species described as *Misturatosphaeria* and *Curreya* or sufficient other genera to be certain about it.
  The hamathecial threads we call pseudoparaphyses in the descriptions, and which have the same width as the paraphyses, may have developed from the latter by elongation and anastomosing, i.e. they may not be pseudoparaphyses. This is, however, difficult to assess. Apically free paraphyses that occur in combination with bitunicate asci also occur in other groups, e.g. the Requienellaceae, Xylariales (Jaklitsch et al. 2016) or the Valsariales (Jaklitsch et al. 2015).
7. Asci: included here for completeness. Asci of the studied species of *Teichospora* are conserved, i.e. they are cylindrical or oblong and only rarely ascospores are partly biseriately arranged; walls are narrow and the endotunica swells in KOH.
8. Ascospores: Colour, shape and septation were used to define genera in the Floricolaceae. There is some correlation of ascospore traits and phylogenetic groups in *Teichospora*. As a genus may contain several species, there must be a phylogenetic substructure and it is to be expected that species with common traits such as ascospore morphology or ecology form subgeneric clusters. As other morphological characters (see above) do not provide arguments for a separation of species at the generic level, we take a look into ascospore morphology and give comments using three examples: Symmetric muriform brown ascospores occur in *T. trabicola* and *T. pusilla*, but also in *Neocurreya* and *Pseudomisturatosphaeria*. Although there is a clear tendency of *T. mariae* to have asymmetric ascospores, a distinction between *Asymmetrispora* and *Pseudomisturatosphaeria* by ascospores with a somewhat tapering lower end and rounded lower end is not always possible, as nearly symmetric ellipsoid ascospores with a broadly rounded lower end are also present in *T. mariae*, the type species of *Asymmetrispora*, depending on the specimen (not shown). *Ramusculicola* is keyed out by Thambugala et al. (2015) as having immersed ascomata; in their notes to the genus, however, *Ramusculicola* is characterised by semi-immersed to partially erumpent ascomata (as also shown by their fig. 30b and c). Otherwise, hyaline didymospores also occur in *T. kenyensis* (*Pseudoaurantiascoma*) and *T. parva* (*Aurantiascoma*). Fungi with hyaline didymospores (but actually all other types of ascospores) are now distributed in several other families of the Pleosporales and other orders of the Dothideomycetes, owing to molecular phylogenetic analyses.
  Taking all data together, morphological characters are at present insufficient to define genera within the Teichosporaceae. However, the main basis for splitting into genera by Thambugala et al. (2015) was the subgeneric structure of *Teichospora* (syn. *Misturatosphaeria*), which calls for a discussion of molecular data and the phylogenetic tree of the Teichosporaceae.

### Evaluation of molecular phylogenetic data

Phylogenetic topologies are subject to changes. The matrix of the “Floricolaceae” used by Thambugala et al. (2015) to compute their phylogenetic tree is basically the same as that used by Mugambi and Huhndorf (2009), and is only augmented by sequences of three taxa recently described in *Curreya*, by *Floricola* spp. and by *Ramusculicola thailandica*. In their tree, many nodes of the backbone receive low or insignificant support. Also, their matrix is inevitably incomplete, as, for the former *Misturatosphaeria* species, it contains only sequences of LSU and *tef1* exon, i.e. markers of low variability, which commonly do not resolve well at the subgeneric level. Unfortunately, it is difficult to determine additional sequences for species described by Mugambi and Huhndorf (2009), because they did not produce cultures but extracted DNA for sequencing from ascomata on the natural specimens. If markers with higher variability such as ITS, *tef1* introns and *rpb2* were added for all taxa, the topology may change substantially, especially in the many nodes with low support, but this may also be the case when additional taxa are added. This is already evident when comparing our phylogenetic tree (Fig. 1) to the tree of Thambugala et al. (2015), which shows different topologies within *Teichosporaceae* likely due to additional taxa as well as sequences (*rpb2*) in our matrix. The addition of *Teichospora trabicola* and *T. pusilla* makes their genus *Neocurreya* paraphyletic, necessitating transfer of *Neocurreya* to *Teichospora*.

In their discussion, Thambugala et al. (2015) wrote “In this paper we introduce 20 new genera which may appear excessive. However, we have only introduced new genera that have been resolved by molecular data and that have further support from morphological data. If we consider that only a small percentage of fungi have been discovered (Hawksworth 1991) and that tropical and saprobic fungi are understudied (Hyde et al. 2010), it is not surprising that there are large numbers of undiscovered taxa”. We agree that the introduction of these genera is excessive, and we do not follow their concept. Their argument of high numbers of undiscovered fungal taxa in the context of new genera is misleading, as generic classification and circumscription have nothing to do with undescribed species biodiversity, which is the topic of Hawksworth (1991). In contrast to species which are (or at least should be) biologically defined entities, genera are artificial units which primarily enable species classification within a binomial classification frame. There is a broadly accepted consensus that genera should be based on monophyly as well as diagnostic features enabling applicable generic circumscription. However, this requires robust multi-gene phylogenies, extensive taxon sampling and thorough investigation of characters. As outlined above, three of the six important criteria of Vellinga et al. (2015) to be considered before the introduction of new fungal genera, viz. sufficient taxon coverage, strong statistical support of the phylogenetic trees and trees based on several genes, are currently not fulfilled in Teichosporaceae, which in our view precludes a persistent generic splitting. As discussed above, many of the characters used by Thambugala et al. (2015) for generic delimitation are problematic as they can be highly variable within species or even specimens. Considering all morphological and molecular phylogenetic evidence at hand, we argue for a broad generic concept to be applied in Teichosporaceae.

### Asexual morphs

The asexual morphs reported as *Coniothyrium*, *Phoma*, *Aposphaeria* and many other genera are only variants of the same principle, i.e. simple pycnidia, which do not offer many characters that may be useful for classification. As an example, *Aposphaeria* Sacc. differs from *Phoma*, which was recently split into several genera by Chen et al. (2015), according to the protologue (Saccardo 1880) in superficial papillate pycnidia. He listed *Melanomma* and *Cucurbitaria* as sexual connections. Samuels and Müller (1979) described the asexual morph of *M. radicans* from artificial culture, i.e. the character superficial versus immersed cannot be assessed, but generally this is not a good generic criterion, as is the formation of apical papillae. Furthermore, colouration of conidia may be slow in certain species, and the hyaline conidia described may turn brown. In any case, currently morphological characters do not receive much attention and genera are based on phylogenetic clades. There is obviously no problem in accepting species with different types of conidia in one genus; see, e.g., *Paracamarosporium* (Crous et al. 2015b). The occurrence of these simple morphs in various unrelated lineages of Dothideomycetes indicates that there is a general genetic potential to produce these asexual morphs of low morphological complexity, i.e. that they may be an ancestral condition or that they can easily evolve independently from the same genetic background. Therefore, their expression in certain genera or species may bear little phylogenetic information, limiting their usability for generic delimitation. The production of minute 1-celled conidia is less energy-consuming than the formation of a complex structure, and thus they may be the principal asexual morphs, while the morph with the larger brown phragmoconidia may be a synanamorph as a result of adaptation to certain niches of the floricola-like species, where the more primitive form may have been lost during evolution.

### Ecology

All species of *Teichospora* are saprotrophs. The majority of species, including the generic type as well as those formerly classified within *Misturatosphaeria*, occur on old wood and bark. However, two of the four species formerly placed in *Curreya* (Marincowitz et al. 2008; Crous et al. 2015b), *T. acaciae* and *T. grandicipis*, are leaf-inhabiting. *Teichospora* (*Floricola*) *striata* was described from senescent leaves and inflorescences of *Juncus roemerianus* (Kohlmeyer and Volkmann-Kohlmeyer 2000), and *T*. (*Floricola*) *viticola* occurs on branches of *Vitis* (Ariyawansa et al. 2015). There is no correlation of these ecological traits with phylogenetic relationships.
